# Success of conservative therapy for chronic subdural hematoma patients: a systematic review

**DOI:** 10.3389/fneur.2023.1249332

**Published:** 2023-09-15

**Authors:** M. Foppen, Harssh Verdan Bandral, Kari-Anne Mariam Slot, W. P. Vandertop, D. Verbaan

**Affiliations:** ^1^Department of Neurosurgery, Amsterdam UMC Location University of Amsterdam, Amsterdam, Netherlands; ^2^Amsterdam Neuroscience, Neurovascular Disorders, Amsterdam, Netherlands

**Keywords:** hematoma, subdural, chronic, humans, treatment outcome, incidence, risk factors

## Abstract

**Background:**

Conservative therapy for chronic subdural hematoma (cSDH) is an option for patients who express no, or only mild symptoms, thereby preventing surgery in some. Because it is not clear for whom conservative therapy is successful, we aimed to estimate the success rate of conservative therapy and to identify which factors might influence success.

**Methods:**

We systematically searched MEDLINE and EMBASE databases to identify all available publications reporting outcome of conservative therapy for cSDH patients. Studies containing >10 patients were included. The primary outcome was the success rate of conservative therapy, defined as “no crossover to surgery” during follow-up. In addition, factors possibly associated with success of conservative therapy were explored. Bias assessment was performed with the Newcastle Ottowa Scale and the Cochrane risk-of-bias tool. We calculated pooled incidence and mean estimates, along with their 95% confidence intervals (CIs), using OpenMeta[Analyst] software.

**Results:**

The search yielded 1,570 articles, of which 11 were included in this study, describing 1,019 conservatively treated patients. The pooled success rate of conservative therapy was 66% (95% CI: 50–82%). One study (*n* = 98) reported smaller hematoma volume to be associated with success, whilst another study (*n* = 53) reported low hematoma density and absence of paresis at diagnosis to be associated with success.

**Conclusion:**

Conservative therapy is reported to be successful in the majority of cSDH patients who have either no, or only mild symptoms. Hematoma volume, low hematoma density and absence of paresis could be factors associated with success. However, further research is warranted in order to establish factors consistently associated with a successful conservative therapy.

**Other:**

No funding was acquired for this study. The study was not registered nor was a study protocol prepared.

## Introduction

Chronic subdural hematoma (cSDH) is a frequently occurring disease mainly affecting elderly patients. Risk factors are brain atrophy, anticoagulation or antiplatelet therapy, male gender and (minor) head trauma (1, 2). The incidence of cSDH is projected to triple by 2040, making its occurrence in daily neurological and neurosurgical practice even more common (3, 4).

There is no consensus concerning the optimal treatment strategy. For patients with severe symptoms (e.g., depressed level of consciousness, hemiparesis, Intractable headache), surgical therapy is the mainstay of treatment. The most frequently used surgical modality is burr hole craniostomy with subdural or subgaleal drainage and in a minority of cases (15%), a craniotomy or twist-drill craniostomy is performed (5). Surgical evacuation is not without disadvantages as it exposes these, often elderly and frail, patients to concomitant risk of complications such as post-operative intracranial haemorrhage, pneumocephaly, seizures, delirium and pneumonia (up to 15%) (6–8). Hematoma recurrence is another well-known complication that arises in the weeks following surgery in approximately 13% of patients (9–11).

Patients who experience relatively mild, or no symptoms, or who are unfit for surgery, can be treated conservatively (wait-and-watch) (12). The frequency of non-surgical therapy as primary treatment has been rising over the last 30 years, but despite this rising frequency, studies regarding the efficacy and outcome are limited (13). Therefore, vital elements such as success rate (i.e., the ability to avoid surgery with good clinical outcome) and factors associated with success have not yet been established.

The absence of clarity results in uncertain and unsubstantiated decisions regarding optimal treatment and follow-up strategy, leading to considerable practice variation (14–16). In order to elucidate these gaps, we reviewed the literature. The primary aim of this study is to (1) determine the reported success rate of conservative therapy and (2) identify factors possibly associated with success.

## Methods

### Search strategy

For this systematic review, we followed the guidelines stated by the Preferred Reporting Items for Systematic Reviews and Meta-Analyses (PRISMA) checklist. We searched MEDLINE and EMBASE databases with the following terms: “chronic subdural hematoma,” “chronic subdural hygroma,”, “conservative treatment,” “non-surgical,” and “observative and observational treatment.” For the detailed search strategy, see Supplement 1. The search was last executed on May 30th, 2023. No other data filters were applied.

### Study selection

Two independent reviewers (MF and HVB) first individually screened the titles and abstracts and subsequently full-text for eligibility. Studies were included if (1) they contained patients diagnosed with a cSDH; (2) age was >18 years old; (3) initial treatment strategy was conservative. Conservative therapy was defined as: “wait-and-scan” or “wait-and-watch.” Studies were also included if only a subgroup received conservative therapy and data of this group could be reliably extracted. For example, if a subgroup received placebo or no treatment (in placebo controlled drug studies or observational studies). Studies were excluded if (1) initial treatment consisted of surgery, middle meningeal artery embolization, medication, epidural blood patch or abstinent therapy; (2) studies contained less than 10 patients; (3) no distinct data of the conservative group could be distilled (also if the success percentage of the conservative group could not be determined due to a missing denominator); (4) wrong publication type (letter to editors, editorials or studies with repeated study population); (5) the language was other than English; (6) it was explicitly described that cSDH occurred after decompressive craniotomy or craniectomy; (7) the hematoma was located infratentorial or other than along the convexity (for supratentorial hematomas); (8) the full-text version was not available. A third adjudicator (DV) was consulted in the case of any discrepancies between the two initial reviewers regarding the in-or exclusion of studies.

### Outcomes

The primary outcome was success of conservative treatment. Conservative therapy was deemed successful if surgical evacuation for the cSDH was not required during follow-up. Other outcomes included factors associated with success of conservative therapy, mortality, Glasgow Outcome Scale (GOS) score, time until complete hematoma resolution in the success group (confirmed by CT-scan), and time to, and criteria for, crossover to surgery.

### Data collection

Two reviewers (MF and HVB) independently collected the variables of interest. Extracted data items included: article information (title, author, year of publication, study design), study in-and exclusion criteria, the total number of patients treated conservatively, criteria for crossover to surgery, clinical characteristics [age, sex, head trauma, use and possible cessation of anticoagulation or antiplatelet therapy, Glasgow Coma Scale (GCS) or Markwalder Grading Scale (MGS)], radiological parameters (hematoma laterality, presence and amount of midline shift, hematoma thickness and volume), GOS, mortality, follow-up time, percentage of successfully treated patients, time from diagnosis of the cSDH to crossover to surgery and time until complete hematoma resolution as confirmed by CT imaging.

### Risk of bias assessment

Two authors (HVB and DV) independently assessed the methodological quality of the included articles using the revised Cochrane “Risk of Bias” tool for Randomized Clinical Trials (RoB 2.0) and the Newcastle-Ottawa Scale (NOS) for observational studies (17, 18). Any discrepancies were discussed until consensus was reached. The RoB 2.0 assesses bias based on five domains: (1) Randomization process; (2) Deviation from intended interventions; (3) Missing outcome data; (4) Measurement of the outcome; (5) Selection of the reported result. Each study is assigned “low concerns of bias” or “some concerns of bias” per domain. The overall bias grade is based on the scores per domain conform RoB 2.0 criteria (19). The NOS can assign up to nine points across three domains for studies with minimum risk of bias. The domains are as follows: (1) Selection of study groups (maximum of four points); (2) Comparability of groups (maximum of two points); (3) Ascertainment of exposure and outcomes (maximum of three points). However, in the case of some studies—particularly those focusing only on chronic subdural hematoma and not on other types of SDH—an unexposed cohort (i.e., a group without the condition) simply could not exist. For these studies, a maximum of three points could be awarded in the first domain, as it was not possible to include a non-exposed cohort. This adjustment resulted in a maximum overall score of eight, rather than nine, for these studies. We converted the NOS ratings to Agency for Healthcare Research and Quality (AHRQ) terms—poor, fair, good—in line with standard conventions. For studies that could obtain nine points “good quality” was awarded if they had three or four points in the first domain, one or two points in the second domain and two or three points in the third domain. For studies that could obtain eight points “good quality” was awarded if they had two or three points in the first domain, one or two points in the second domain and two or three points in the third domain.

### Statistical analysis

For continuous variables, means and 95% confidence intervals (CI) were calculated for all patients treated with conservative therapy. For dichotomous outcomes, a pooled estimate with 95% CI was calculated. All statistical analyses were performed using OpenMeta[Analyst] (CEBM, Brown University, 2012) (20).

## Results

### Search

**Figure 1:**
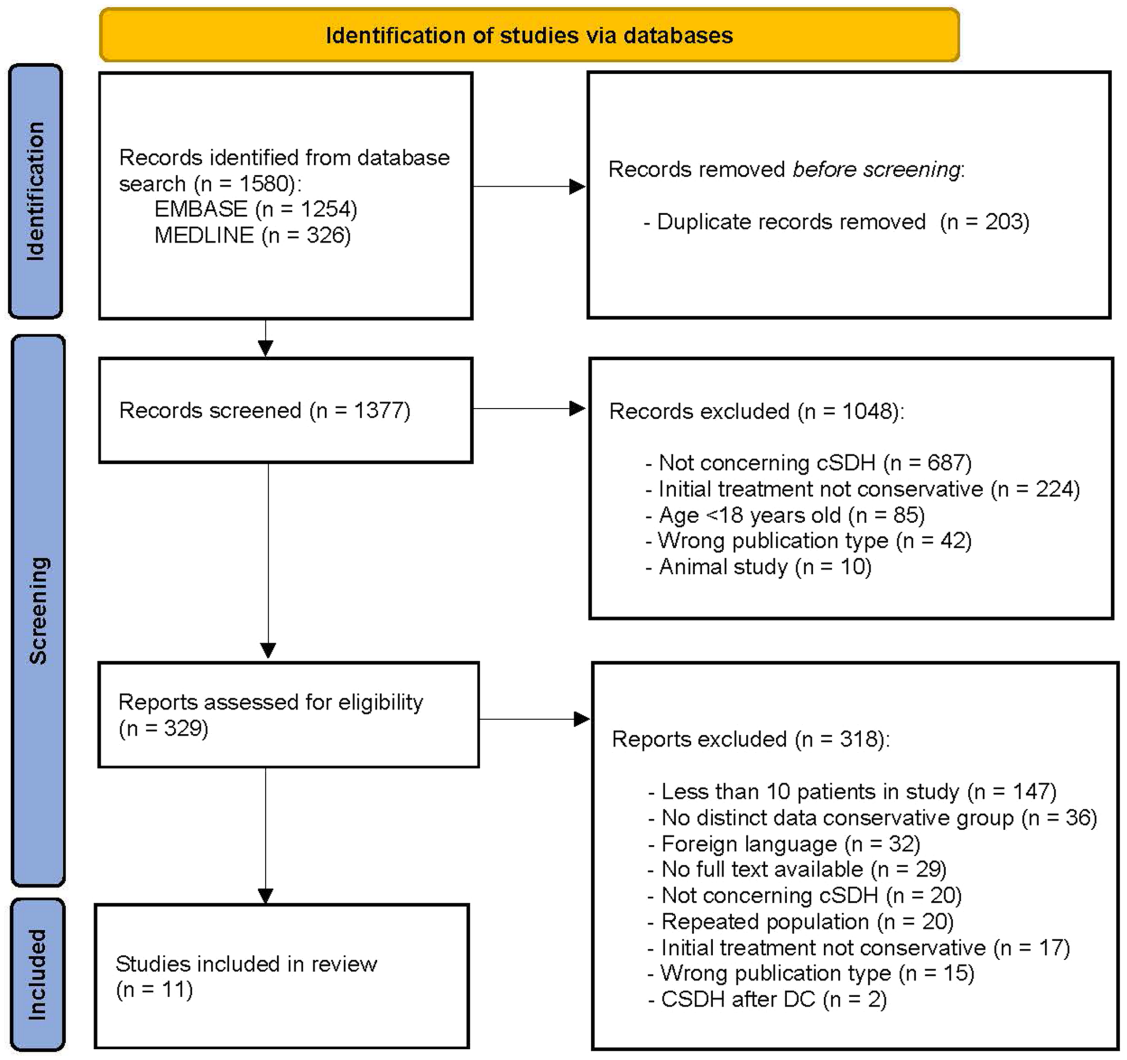
PRISMA flow diagram of study selection process (21). DC, decompressive craniectomy.

The initial search yielded 1,570 studies. After removing duplicates and screening the title and abstract 329 full-text articles were assessed for eligibility. Upon reviewing the full-text articles, a total of 11 studies were included (Figure 1) (21).

### Study characteristics

Of the 11 included studies, six were retrospective cohort studies, two prospective cohort studies, one a pilot RCT, one a RCT and one a *post-hoc* analysis of a previous RCT. The date of publication ranged from 1974 to 2022. Two cohort studies provided a direct comparison between patients treated successfully with conservative therapy and patients for whom conservative therapy had failed (22, 23). In the pilot RCT the effect of dexamethasone was compared to placebo and in the RCT the effect of etizolam was compared to no treatment (24, 25). The study of Wang et al. (22), was a *post-hoc* analysis of an RCT in which the effect of atorvastatin vs. placebo was investigated (22, 26). The mean follow-up duration of all studies was 4.6 months. See Table 1 for study characteristics.

**Table 1:** Included studies, study characteristics and outcomes.

Study	Study type	*N*	Success (%)	Time till crossover	Time till complete resolution on CT	Mortality	Follow-up	Glasgow outcome scale (%)
Bender et al. (27)	R	60	48 (80)	NA	NA	NA	30 months	NA
Hirashima et al. (25)	RCT	29	2 (6.9)	NA	NA	NA	6 months	NA
Prud'homme et al. (24)	Pilot RCT	10	7 (70)	NA	24 weeks	0 (0)	6 months	NA
Kim et al. (23)	R	16	13 (81.3)	39 days	17 weeks (4–96 weeks)	0 (0)	Until nearly complete hematoma resolution	5 (100)
Chan et al. (28)	R	12	5 (41.7)	NA	NA	NA	6 months	5 (17) 4 (83)
Asan et al. (29)	R	163	133 (85.6)	NA	NA	NA	23 days	NA
Hou et al. (30)	P	26	26 (100)	NA	10 weeks	0 (0)	73 days	NA
Ban et al. (31)	P	67	11 (16.4%)	NA	NA	NA	6 months	NA
Rauhala et al. (13)	R	223	170 (76.2)	24 days	NA	NA	Minimally 24 months or until death	NA
Petralia et al. (32)	R	315	293 (93.0)	NA	NA	NA	1 month	NA
Wang et al. (22)	*Post-hoc* RCT analysis	98	75 (76.5)	25 days	NA	0 (0)	6 months	5 (67) 4 (10) 3 (23)^§^
Total n*		1,019	783/1,019 (76.8%)	25 days (*n* = 79)	14.7 weeks (*n* = 49)	0/150 (0.0%)	4.6 months (*n* = 780)	5 (66) 4 (16) 3 (17)
Pooled estimate (95% CI)			66.0% (49.7–82.3)			0.0 (0.0–2.0)		

R, retrospective cohort study; P, prospective cohort study; RCT, randomized controlled trial; NA, not available; CI, confidence interval. *Averages were calculated were possible. For the outcomes time till crossover, time till complete resolution and follow-up no 95% CI could be calculated since measures of dispersion were not provided. ^§^The GOS was determined 8 weeks after diagnosis in 88 patients.

### Risk of bias assessment

**Figure 2:**
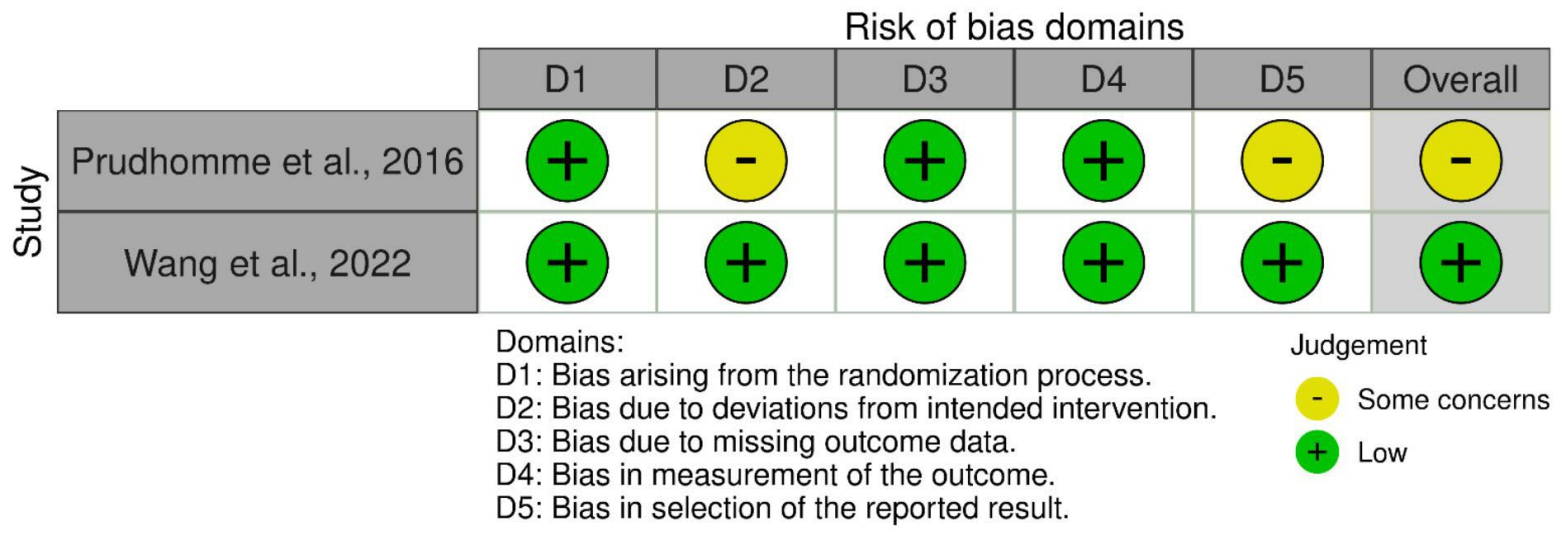
Risk of bias and quality assessment of randomized studies with the Risk of Bias 2.0.

The AHRQ quality was “poor” in seven of the nine included cohort studies. The quality was “good” in the studies by Hirashima et al. (25) and by Rauhala et al. (13). The risk of bias per domain, as assessed by the NOS, is summarized for each study in Table 2. Among the two randomized studies, one had a low risk of bias, while the other raised some concerns (Figure 2).

**Table 2:** Risk of bias and quality assessment for observational studies with NOS-scale.

Study	Selection	Comparability	Exposure	Quality
Bender et al. (27)	★		★★	Poor
Hirashima et al. (25)	★★	★★	★★★	Good
Kim et al. (23)	★★		★★	Poor
Chan et al. (28)	★★		★★★	Poor
Asan et al. (29)*	★★★★		★★	Poor
Hou et al. (30)	★		★★	Poor
Ban et al. (31)		★★	★★★	Poor
Rauhala et al. (13)	★★★	★	★★★	Good
Petralia et al. (32)	★		★★	Poor

The last column indicates AHRQ quality standards. *Was only study that could be assigned nine stars due to its methodology.

### Patient characteristics

All studies contained a total of 1,019 patients treated with conservative therapy. The mean age was 66.8 years (95% CI: 64.5–69.2, *n* = 71,) and the population was predominantly male (82%, 95% CI: 70.9–90.6%, *n* = 191) (Table 3). The Glasgow Coma Scale was reported for 369 patients, of which 353 had scores between 14-15 (95.7%) (23, 28, 30, 32). The Markwalder Grading Scale was reported for 196 patients, with 136 scoring between 0 and 2 (69.4%) (22–24, 27, 28). The mean midline shift was 4.6 millimeters (95% CI: 2.0–9.8 millimeters) in 124 patients and the mean hematoma thickness was 15.9 millimeters (95% CI: 13.7–18.0) in 124 patients (22–24). In 114 patients, hematoma volume was reported and had a mean of 55.2 milliliters (95% CI: 38.8–72.4 milliliters) (22, 23).

**Table 3:** Demographical, clinical and radiological characteristics of patients per study.

Study	Age	Male (%)	Head trauma (%)	AC/AP use (%)	GCS	MGS	Unilateral (%)	Midline shift (%)	Midline shift (mm)	Hematoma thickness (mm)	Hematoma volume (ml)
Bender et al. (27)	NA	NA	NA	NA	NA	0–3	NA	NA	NA	NA	NA
Hirashima et al. (25)	68.1 (8.6)	22 (75.9)	25 (100)	NA	NA	NA	15 (51.7)	NA	NA	NA	NA
Prud'homme et al. (24)	69.4 (8.8)	10 (100)	7 (70)	8 (80)	NA	0–2	NA	NA	8.0 (3.4)	20.4 (6.1)	NA
Kim et al. (23)	64.7 (16.9)	11 (68.8)	13 (81.2)	3 (18.8)	>8	0–2	13 (81.2)	12 (75)	7.3 (4.6)	14.2 (3.0)	46.2 (17.4)
Chan et al. (28)	79.5 (58–95)	7 (58.3)	NA	6 (50)	14	0–2	12 (100)	NA	2 (1–4)	13 (7.8–21)	41.4 (25–65)
Asan et al. (29)	NA	NA	NA	NA	NA	NA	NA	NA	NA	NA	NA
Hou et al. (30)	64.4 (9.6)	19 (73.1)	26 (100)	0 (0)	15	NA	17 (65.4)	NA	<5 mm	NA	NA
Ban et al. (31)	NA	NA	NA	NA	NA	NA	NA	67 (100)	>10 mm	NA	NA
Rauhala et al. (13)	NA	NA	NA	NA	NA	NA	NA	NA	NA	NA	NA
Petralia et al. (32)	NA	NA	NA	NA	14–15	NA	NA	NA	<3 mm	NA	NA
Wang et al. (22)	67 (26–89)	88 (89.8)	NA	NA	NA	0–2	61 (62.2)	NA	2.7 (3.5)	15.3 (5.4)	63.8 (33.9)
Total *n* (%)	71	157/191 (78.1)	71/81 (95.5)	17/64 (26.6)			118/181 (65.7)	79/83 (95.2)	124	124	114
Pooled estimate (CI)^¥^	66.8 (64.5–69.2)	82.0 (70.9–90.6)	87.7 (76.6–99.6)	36.1 (1.6–70.7)			71.8 (54.5–89.0)	89.5 (66.2–100)	4.6 (2.0–9.8)	15.9 (13.7–18.0)	55.2 (38.0–72.4)

NA, not available; AC, anticoagulant; AP, antiplatelet; GCS, Glasgow Coma Scale; MGS, Markwalder Grading scale; mm, millimeter; ml, milliliter. ^¥^For dichotomous and continuous variables a 95% confidence interval was calculated.

### Outcomes

The success rate of conservative therapy was 66.0% (95% CI: 49.2–82.3%), ranging from 6.9–100% (Table 1). Factors associated with crossover to surgery were evaluated in three studies (Table 4) (22, 23, 25). Larger hematoma volume was a predictor of crossover to surgery in one study (OR 1.019, 95% CI: 1.002–1.037) (22). In another study, hematoma volume was lower in the success group, albeit not significant (43.2 milliliters vs. 62 milliliters, *p* = 0.146) (23). In one study, paresis at diagnosis was associated with crossover to surgery (OR 6.35, 95% CI: 1.04–38.7) and low hematoma density was negatively associated with crossover to surgery (OR: 0.125, 95% CI: 0.01–0.85) (25). Criteria for crossover to surgery were provided in eight studies. The mean period between diagnosis and crossover to surgery was 25 days (*n* = 79) (13, 22, 23). Time until complete resolution of the cSDH was reported in three studies and with a mean of 14.7 weeks (*n* = 46) (23, 24, 30). Mortality was (95% CI: 0.0–2.0%) in 150 cases (22–24, 30). The GOS was reported for 116 patients, of whom 77 had a good recovery (66.4%, GOS 5), 19 had a moderate disability (16.4%, GOS 4) and 20 patients had a severe disability (17.2%, GOS 3) at the end of follow-up (22, 23, 28).

**Table 4:** Indications for conservative therapy, antithrombotic strategy and indications for crossover to surgery.

Study	Indication conservative therapy		AC/AP strategy	Criteria for crossover	Factors associated with success or crossover
	Inclusion	Exclusion			
Bender et al. (27)	NA	NA	NA	NA	NA
Harishima et al. (25)	If patient could walk and eat without help	Massive hematoma, impeding sign of brain herniation, severe headache, vomiting, paresis or complications (cardiopulmonary, hepato-renal or metabolic)	NA	Increase in hematoma size and aggravation of symptoms	Low density hematoma*, paresis at diagnosis**
Prud'homme et al. (24)	MGS 0-2	Cranial surgery in last year, if AC therapy could not be stopped for 6 months	NA	Sudden increase in hematoma volume, midline shift >10 mm, deterioration of level of consciousness	NA
Kim et al. (23)	Hematoma that exerts mass effect, mild symptoms	GCS < 8	NA	Newly or progressive neurological deficits	No significant factors found
Chan et al. (28)	MGS 0-2, GCS 14-15	No immediate surgery indication, cSDH secondary to underlying hematological disorder or malignancy	Stopped during period of conservative therapy	Any worsening of symptoms, GCS decrease, new focal neurological deficits or radiological hematoma progression	NA
Asan et al. (29)	NA	NA	NA	NA	NA
Hou et al. (30)	Head trauma in 3 months prior to cSDH, midline shift >10 mm, GCS 15, no evidence of intracranial hypertension	Coagulopathy or AC/AP use, predisposing diseases to cSDH	Patients with AP/AC use excluded from study	Enlargement of cSDH, progressive aggravation of the neurologic deficit and signs of intracranial hypertension	NA
Ban et al. (31)	>20 years old, asymptomatic	CSDH due to underlying condition, shift <10 mm or no mass effect	NA	Occurrence of symptoms and/or increase in hematoma thickness	NA
Rauhala et al. (13)	No significant neurological symptoms	NA	Discontinued at diagnosis	Increase in cSDH size	NA
Petralia et al. (32)	GCS 14-15, shift <3 mm	No other intracranial bleeds (<20cm^3^)	NA	NA	NA
Wang et al. (22)	No risk of cerebral herniation, MGS/GCS < 3	Antiplatelet medication	Excluded patients with AP, no comment on patients with AC	Neurologic function deterioration, radiological hematoma progression or > 1 cm shift	Hematoma volume*

The most notable in-and exclusion criteria per study are provided. *Associated with crossover to surgery;**Negatively associated with crossover to surgery. AC, anticoagulant therapy; AP, antiplatelet therapy.

## Discussion

This systematic literature review shows that the mean success rate of conservative treatment for cSDH patients with no, or only mild symptoms is reported to be 66%. Hematoma volume, low hematoma density and absence of paresis at diagnosis could be factors associated with success of conservative therapy.

This study demonstrates that the success rate of a wait-and-scan, or a wait-and-watch, strategy can be quite high in a selected group of patients with cSDH. Although success is primarily defined as “no need for surgery,” true success should of course be defined as good clinical outcome. However, specific and reliable data are usually lacking. Nevertheless, the range of successful conservative strategy varied greatly in all studies. This could largely be attributed to study heterogeneity regarding indication for conservative therapy and applied in-and exclusion criteria. Studies including patients with larger hematomas reported a higher crossover rate, whereas studies with smaller hematomas tended to report a lower crossover rate (31, 32).

Interestingly, conservative therapy can also be successful in patients with noteworthy clinical expression of their cSDH (e.g., patients with a Markwalder score of 1 or 2). This raises the question of whether it would be justified, and potentially beneficial, to postpone and withhold surgery in more patients than currently is being practiced in standard care. If so, an unnecessary number of patients could well be exposed to anesthetic and surgical risks by not considering conservative therapy more often. Vice versa, evaluating the success rate of conservative therapy in asymptomatic patients (e.g., patients with a Markwalder score of 0) would also be interesting as the success rate is potentially higher for these patients than found in this study. In a recent study by Parry et al. (33) the crossover to surgery rate was determined in a highly pre-selected asymptomatic cohort of 106 cSDH patients (all Markwalder score 0) receiving conservative therapy. Only one patient (0.9%) required neurosurgical intervention within three months after diagnosis. In our study we could not determine success rate stratified per Markwalder scale, as it was not reported in such detail. More prospective research is required to provide insight into this matter.

In our study we also assessed potential factors associated with success of conservative therapy. Yet, there was a lack of consistency, as none of the described factors were associated with success across multiple studies. In fact, hematoma volume was associated with success in the study by Wang et al. (22), but not in the study of Kim et al. (23). Hence, factors associated with success have to be investigated more thoroughly before they can aid clinical decision making in the future. Nowadays, physicians are still unable to identify which hematomas will resolve spontaneously and which will progress to become symptomatic. This implies that every patient is to be followed with similar caution since it is not possible to distinguish potential surgical candidates from patients who are not.

An important limitation of this review is the risk of selection bias in the included studies. Most studies were retrospective and all included patients were presented to a neurological or neurosurgical department. Thus, some asymptomatic patients (those not seeking medical attention) are missed. This arguably resulted in an underestimation of the true success rate. Also, the indication for crossover to surgery differed between most studies and was rather subjective and inevitable since specific criteria for crossover are not available. This certainly influenced the primary outcome of this study. Although to what extent this resulted in over-or underestimation of the crossover rate is not clear, since preference of the attending physicians regarding treatment strategy was not objectified. Finally, the lack of data for other aspects of conservative therapy, especially data concerning clinical outcome, precluded assessing the overall effect of conservative therapy. Therefore, no definite conclusions about clinical outcome or indication of conservative therapy can be drawn.

In order to provide more high-quality evidence regarding the effect of conservative therapy for cSDH patients additional research is required. Ideally, such future studies are prospective and multicenter, and a joint venture of neurological and neurosurgical departments due to the nature of this disease and its treatment paradigm. Furthermore, rigorous data regarding clinical outcome are to be incorporated in future studies. With regard to outcomes of conservative therapy of future studies we recommend using the results of the Delphi-survey of the CODE-CSDH project when available (34). This consortium aims to establish core outcomes for cSDH, thereby preventing heterogeneity in this field of research (35).

## Conclusion

Success of a wait-and-scan, or a wait-and-watch, strategy is reported to be quite high in the majority of a selected group of patients with cSDH. We could not establish any consistent factors that influence success of conservative therapy. Due to the high risk of selection bias in existing literature, the absence of high-quality methodological studies and the scarcity of available data, further research regarding outcome of conservative therapy is necessary to establish its definite place and value in cSDH treatment.

## Data availability statement

The original contributions presented in the study are included in the article/Supplementary material, further inquiries can be directed to the corresponding author.

## Author contributions

MF, DV, and WV initiated the study and designed the research plan. MF and HB performed the data selection, data analysis, and drafted the manuscript. DV and HB performed the quality assessment of included studies. K-AMS, DV, and WV critically revised the final manuscript before submission. All authors contributed to the article and approved the submitted version.

## Conflict of interest

The authors declare that the research was conducted in the absence of any commercial or financial relationships that could be construed as a potential conflict of interest.

## Publisher’s note

All claims expressed in this article are solely those of the authors and do not necessarily represent those of their affiliated organizations, or those of the publisher, the editors and the reviewers. Any product that may be evaluated in this article, or claim that may be made by its manufacturer, is not guaranteed or endorsed by the publisher.

## References

[1] Yang W Huang J. Chronic subdural hematoma: epidemiology and natural history. Neurosurg Clin N Am. (2017) 28:205–10. doi: 10.1016/j.nec.2016.11.002 28325454

[2] Sahyouni R Goshtasbi K Mahmoodi A Tran DK Chen JW. Chronic subdural hematoma: a historical and clinical perspective. World Neurosurg. (2017) 108:948–53. doi: 10.1016/j.wneu.2017.09.064, PMID: 28935548 PMC12747318

[3] Stubbs DJ Vivian ME Davies BM Ercole A Burnstein R Joannides AJ. Incidence of chronic subdural haematoma: a single-Centre exploration of the effects of an ageing population with a review of the literature. Acta Neurochir. (2021) 163:2629–37. doi: 10.1007/s00701-021-04879-z, PMID: 34181085 PMC8357776

[4] Kolias AG Chari A Santarius T Hutchinson PJ. Chronic subdural haematoma: modern management and emerging therapies. Nat Rev Neurol. (2014) 10:570–8. doi: 10.1038/nrneurol.2014.163, PMID: 25224156

[5] Cenic A Bhandari M Reddy K. Management of chronic subdural hematoma: a national survey and literature review. Can J Neurol Sci. (2005) 32:501–6. doi: 10.1017/S0317167100004510, PMID: 16408582

[6] Pang CH Lee SE Kim CH Kim JE Kang HS Park CK . Acute intracranial bleeding and recurrence after bur hole craniostomy for chronic subdural hematoma. J Neurosurg. (2015) 123:65–74. doi: 10.3171/2014.12.JNS141189, PMID: 25679282

[7] Rohde V Graf G Hassler W. Complications of burr-hole craniostomy and closed-system drainage for chronic subdural hematomas: a retrospective analysis of 376 patients. Neurosurg Rev. (2002) 25:89–94. doi: 10.1007/s101430100182, PMID: 11954771

[8] Chen FM Wang K Xu KL Wang L Zhan TX Cheng F . Predictors of acute intracranial hemorrhage and recurrence of chronic subdural hematoma following burr hole drainage. BMC Neurol. (2020) 20:92. doi: 10.1186/s12883-020-01669-5, PMID: 32169039 PMC7069197

[9] Almenawer SA Farrokhyar F Hong C Alhazzani W Manoranjan B Yarascavitch B . Chronic subdural hematoma management: a systematic review and meta-analysis of 34,829 patients. Ann Surg. (2014) 259:449–57. doi: 10.1097/SLA.0000000000000255 24096761

[10] Henry J Amoo M Kissner M Deane T Zilani G Crockett MT . Management of Chronic Subdural Hematoma: a systematic review and component network Meta-analysis of 455 studies with 103 645 cases. Neurosurgery. (2022) 91:842–55. doi: 10.1227/neu.0000000000002144, PMID: 36170165

[11] Lodewijkx R Foppen M Slot KM Vandertop WP Verbaan D. Recurrent chronic subdural hematoma after burr-hole surgery and postoperative drainage: a systematic review and meta-analysis. Operative neurosurgery (Hagerstown, Md.). (2023). 25, 216–241. doi: 10.1227/ons.0000000000000794 37387582 PMC10389757

[12] Soleman J Nocera F Mariani L. The conservative and pharmacological management of chronic subdural haematoma. Swiss Med Wkly. (2017) 147:w14398. doi: 10.57187/smw.2017.14398, PMID: 28102879

[13] Rauhala M Helén P Huhtala H Heikkilä P Iverson GL Niskakangas T . Chronic subdural hematoma-incidence, complications, and financial impact. Acta Neurochir. (2020) 162:2033–43. doi: 10.1007/s00701-020-04398-3, PMID: 32524244 PMC7415035

[14] Berghauser Pont LM Dippel DW Verweij BH Dirven CM Dammers R. Ambivalence among neurologists and neurosurgeons on the treatment of chronic subdural hematoma: a national survey. Acta Neurol Belg. (2013) 113:55–9. doi: 10.1007/s13760-012-0130-1, PMID: 22975837

[15] Holl DC Blaauw J Ista E Dirven CMF Kho KH Jellema K . National survey on the current practice and attitudes toward the management of chronic subdural hematoma. Brain Behav. (2022) 12:e2463. doi: 10.1002/brb3.2463, PMID: 35113493 PMC8933788

[16] Laldjising ERA Cornelissen FMG Gadjradj PS. Practice variation in the conservative and surgical treatment of chronic subdural hematoma. Clin Neurol Neurosurg. (2020) 195:105899. doi: 10.1016/j.clineuro.2020.105899, PMID: 32516640

[17] BS GA Wells O'Connell D Peterson J Welch V Losos M Tugwell P. The Newcastle-Ottawa scale (NOS) for assessing the quality of nonrandomised studies in meta-analyses. (2021). Available at: https://www.ohri.ca/programs/clinical_epidemiology/oxford.asp.

[18] Sterne JAC Savović J Page MJ Elbers RG Blencowe NS Boutron I . RoB 2: a revised tool for assessing risk of bias in randomised trials. BMJ. (2019) 366:l4898. doi: 10.1136/bmj.l4898 31462531

[19] JPT Higgins Savović J Page M.J. Sterne J.A.C. Revised Cochrane risk-of-bias-tool for randomized trials (RoB 2). (2019). Available at: https://guides.library.uq.edu.au/referencing/vancouver/webpages (Accessed 13 June, 2023).

[20] Wallace BC Dahabreh IJ Trikalinos TA Lau J Trow P Schmid CH. Closing the gap between methodologists and end-users: R as a computational Back-end. J Stat Softw. (2012) 49:1–15. doi: 10.18637/jss.v049.i05

[21] Page MJ McKenzie JE Bossuyt PM Boutron I Hoffmann TC Mulrow CD . The PRISMA 2020 statement: an updated guideline for reporting systematic reviews. BMJ. (2021) 372:n71. doi: 10.1136/bmj.n71 33782057 PMC8005924

[22] Wang D Tian Y Wei H Gao C Fan Y Yang G . Risk factor analysis of the conservative treatment in chronic subdural hematomas: a substudy of the ATOCH trial. Adv Ther. (2022) 39:1630–41. doi: 10.1007/s12325-022-02057-w, PMID: 35133631

[23] Kim HC Ko JH Yoo DS Lee SK. Spontaneous resolution of chronic subdural hematoma: close observation as a treatment strategy. J Korean Neurosurg Soc. (2016) 59:628–36. doi: 10.3340/jkns.2016.59.6.628, PMID: 27847578 PMC5106364

[24] Prud'homme M Mathieu F Marcotte N Cottin S. A pilot placebo controlled randomized trial of dexamethasone for chronic subdural hematoma. Can J Neurol Sci. (2016) 43:284–90. doi: 10.1017/cjn.2015.393, PMID: 26853325

[25] Hirashima Y Kurimoto M Nagai S Hori E Origasa H Endo S. Effect of platelet-activating factor receptor antagonist, etizolam, on resolution of chronic subdural hematoma--a prospective study to investigate use as conservative therapy. Neurol Med Chir (Tokyo). (2005) 45:621–6; discussion 6. doi: 10.2176/nmc.45.621, PMID: 16377949

[26] Jiang R Zhao S Wang R Feng H Zhang J Li X . Safety and efficacy of atorvastatin for chronic subdural hematoma in Chinese patients: a randomized ClinicalTrial. JAMA Neurol. (2018) 75:1338–46. doi: 10.1001/jamaneurol.2018.2030, PMID: 30073290 PMC6248109

[27] Bender MB Christoff N. Nonsurgical treatment of subdural hematomas. Arch Neurol. (1974) 31:73–9. doi: 10.1001/archneur.1974.00490380021001 4834968

[28] Chan DY Chan DT Sun TF Ng SC Wong GK Poon WS. The use of atorvastatin for chronic subdural haematoma: a retrospective cohort comparison study(). Br J Neurosurg. (2017) 31:72–7. doi: 10.1080/02688697.2016.1208806, PMID: 27881024

[29] Asan Z. Growth potential of subdural hematomas under clinical observation: which subdural hematomas tend to grow and why they do. World Neurosurg. (2018) 113:e598–603. doi: 10.1016/j.wneu.2018.02.106, PMID: 29486314

[30] Hou K Zhu X Zhao J Zhang Y Gao X Jiang K . Efficacy of reinforced restriction of physical activity on chronic subdural hematoma: prospective pilot study. World Neurosurg. (2018) 110:e1011–6. doi: 10.1016/j.wneu.2017.11.155, PMID: 29223519

[31] Ban SP Hwang G Byoun HS Kim T Lee SU Bang JS . Middle meningeal artery embolization for chronic subdural hematoma. Radiology. (2018) 286:992–9. doi: 10.1148/radiol.2017170053 29019449

[32] Petralia CCT Manivannan S Shastin D Sharouf F Elalfy O Zaben M. Effect of steroid therapy on risk of subsequent surgery for neurologically stable chronic subdural hemorrhage-retrospective cohort study and literature review. World Neurosurg. (2020) 138:e35–41. doi: 10.1016/j.wneu.2020.01.160, PMID: 32113994

[33] Parry D Baskaran R Lima A Dagnan R Jaber H Manivannan S . Asymptomatic chronic subdural haematoma - does it need neurosurgical intervention? Br J Neurosurg. (2023) 1-6:1–6. doi: 10.1080/02688697.2023.2210224 37237434

[34] Holl DC Chari A Iorio-Morin C Dammers R van der Gaag NA Kolias AG . Study protocol on defining Core outcomes and data elements in chronic subdural Haematoma. Neurosurgery. (2021) 89:720–5. doi: 10.1093/neuros/nyab268, PMID: 34318894 PMC8440066

[35] Chari A Hocking KC Broughton E Turner C Santarius T Hutchinson PJ . Core outcomes and common data elements in chronic subdural hematoma: a systematic review of the literature focusing on reported outcomes. J Neurotrauma. (2016) 33:1212–9. doi: 10.1089/neu.2015.3983, PMID: 26295586 PMC4931358

