# Supplementary material for: Success of conservative therapy for chronic subdural hematoma patients: a systematic review
Source: Front Neurol. 2023 Sep 15;14:1249332. doi: 10.3389/fneur.2023.1249332 (PMC10540204; doi:10.3389/fneur.2023.1249332)
Supplement: Supplementary file 1 [file Table_1.DOCX]

# Supplement 1: Online search strategy

| Database(s): Ovid MEDLINE(R) ALL 1946 to May 30, 2023 | |  |
| --- | --- | --- |
| Search Strategy: | |  |
| # | Searches | Results |
| 1 | exp Hematoma, Subdural, Chronic/ | 1799 |
| 2 | ((chronic adj3 (subdural adj3 (h?ematoma* or h?emorrhage or bleed* or blood))) or cSDH).ti,ab,kf. | 3698 |
| 3 | (subdural adj3 hygroma).ti,ab,kf. | 507 |
| 4 | or/1-3 | 4254 |
| 5 | exp Conservative Treatment/ | 4932 |
| 6 | ((non or "not") adj3 (operat* or therap* or treat* or surg*)).ti,ab,kf. | 419855 |
| 7 | ((conservative or nonsurg* or nonoperat* or "non surg*" or "non operat*") adj3 (therap* or treat* or manag* or observ*)).ti,ab,kf. | 108311 |
| 8 | (wait adj3 (watch* or see* or observ* or scan)).ti,ab,kf. | 3695 |
| 9 | or/5-8 | 510530 |
| 10 | 4 and 9 | 326 |

*MEDLINE search*

| Database(s): Embase Classic+Embase 1947 to 2023 May 30 | |  |
| --- | --- | --- |
| Search Strategy: | |  |
| # | Searches | Results |
| 1 | exp subdural hematoma/ | 23535 |
| 2 | (("chronic subdural" adj3 (h?ematoma* or h?emorrhage or bleed* or blood)) or cSDH).ti,ab,kf. | 4649 |
| 3 | (subdural adj3 hygroma).ti,ab,kf. | 719 |
| 4 | or/1-3 | 24346 |
| 5 | exp conservative treatment/ | 744121 |
| 6 | ((non or "not") adj3 (operat* or therap* or treat* or surg*)).ti,ab,kf. | 676907 |
| 7 | ((conservative or nonsurg* or nonoperat* or "non surg*" or "non operat*") adj3 (therap* or treat* or manag* or observ*)).ti,ab,kf. | 157476 |
| 8 | (wait adj3 (watch* or see* or observ* or scan)).ti,ab,kf. | 6175 |
| 9 | or/5-8 | 1461487 |
| 10 | 4 and 9 | 2199 |
| 11 | exp observational study/ or exp observational method/ or exp prognosis/ or exp outcome assessment/ or exp cohort analysis/ or exp longitudinal study/ or exp prospective study/ or exp retrospective study/ or exp Glasgow outcome scale/ or exp treatment outcome/ | 5358664 |
| 12 | ((cohort* or "follow up" or longitudinal or prospective or retrospective) adj3 stud*).ti,ab,kf. | 1820952 |
| 13 | (observ* or prognos* or outcome).ti,ab,kf. | 8093410 |
| 14 | or/11-13 | 11227283 |
| 15 | 10 and 14 | 1254 |

*EMBASE search*
